# Antiproliferative and Apoptotic Effects of *Sesbania grandiflora* Leaves in Human Cancer Cells

**DOI:** 10.1155/2014/474953

**Published:** 2014-05-15

**Authors:** Sankar Pajaniradje, Kumaravel Mohankumar, Ramya Pamidimukkala, Srividya Subramanian, Rukkumani Rajagopalan

**Affiliations:** Department of Biochemistry and Molecular Biology, School of Life Sciences, Pondicherry University, Puducherry 605 014, India

## Abstract

Natural phytochemicals and their derivatives are good drug candidates for anticancer therapeutic approaches against multiple targets. We report here the initial findings from our studies on the anticancer properties of the leaves of the medicinal plant *Sesbania grandiflora*. In the current study, five different solvent fractions from the leaves of *S. grandiflora* were tested on cancer cell lines such as MCF-7, HepG2, Hep-2, HCT-15, and A549. The methanolic fraction of *S. grandiflora* was found to exert potent antiproliferative effects especially in the human lung cancer cell line, A549. Caspase 3 was activated in the methanolic fraction treated A549 cells thereby leading to cell death by apoptosis. DAPI staining, DNA laddering, and decrease in mitochondrial membrane potential further confirmed the apoptotic mode of cell death. The high levels of ROS intermediates as evidenced by DCF-DA staining could have played a role in the apoptotic induction. Decrease in levels of cyclin D1 and decrease in the activation of NFkB were observed in A549 cells on treatment with methanolic fraction, giving a hint on the possible mechanism of action. These results prove that the medicinal plant *S. grandiflora* can be explored further for promising candidate molecules to combat cancer, especially lung cancer.

## 1. Introduction

Natural products have been a continuous source of medicines for a very long time. From ancient days, natural products have been the sole means to treat diseases and injury. In most traditional systems of medicine worldwide, plant-based products serve as an integrated part of treatment. The therapeutic effects of these plants have been subsequently proved by medical practices.

Cancer is a major health hazard worldwide. Cancer treatment relies on chemotherapy using cytotoxic drugs, radiation therapy, and surgery. Today a variety of cytotoxic drugs have been reported to combat cancer. Most of these drugs are inadequate not only because of their therapeutic efficacy but also because they have undesirable side effects. With the aim of searching novel compounds without undesirable side effects, we focused on natural medicines. Plants are reported to have a long history in the treatment of cancer [1]. The use of plants and plant-based products for cancer treatment is rapidly growing in medical practices [2]. This led us to choose the medicinal plant* Sesbania grandiflora* L. This plant has been used in the traditional medicine of the Indian Ayurvedic system. Though already proven to have varied medicinal uses like hepatoprotective and cardioprotective roles [3, 4],* S. grandiflora* is the plant of interest in the last couple of years especially for its chemopreventive effects. Studies have shown that the flowers of this plant have potent anticancer activities in various cancer cell models [5, 6]. In many parts of Southeast Asian countries, the flowers and leaves of* S. grandiflora* are used in medicine as well as in traditional food. It has also been shown that the roots of* S. grandiflora* possess antituberculosis activity [7]. The flower extracts of this plant have also been proved to possess antimicrobial activities [8]. In one of our previous studies we have found that the leaves of this plant possess protective roles against rat kidney during alcohol and polyunsaturated fatty acid induced oxidative stress [3]. The leaves have shown anxiolytic and anticonvulsive activity in experimental rats [9]. The leaf juice also seems to possess antiurolithiatic and antioxidant properties [10]. Though the flowers have now proven to have chemopreventive effects [11], not much work, including mechanism of action and molecular level studies, has been carried out to prove the chemopreventive effects of the* S. grandiflora* leaves. The current study is focused on the antiproliferative effects of the leaves of* S. grandiflora* in human cancer cell models.

## 2. Materials and Methods

### 2.1. Materials

Dulbecco's modified Eagle's medium (DMEM), fetal bovine serum (FBS), rhodamine 123, dichlorofluorescein diacetate (DCF-DA) and 4′,6-diamidino-2-phenylindole (DAPI), and the antibodies for *β*-actin and NF*κ*B were purchased from Sigma (St. Louis, MO, USA). Primary antibodies for cyclin D1 (SC-246), caspase 3 (SC-7148), and lamin B (SC-6217) and secondary antibodies, goat anti-rabbit IgG-HRP (SC-2004), goat anti-mouse IgG-HRP (SC-2005), were purchased from Santa Cruz Biotechnology (Santa Cruz, CA, USA).

### 2.2. Sample Preparation for Cell Culture

The dried leaves of* S. grandiflora* were subjected to solvent extraction using solvents of increasing polarity such as petroleum ether, chloroform, acetone, methanol, and water by using Soxhlet extractor. Each solvent fraction was distilled and concentrated. Water insoluble fractions were concentrated using rotary evaporator and subjected to vacuum under reduced pressure overnight yielding the fractions in powder form. Water soluble fraction was subjected to lyophilization (−80°C, in vacuum under reduced pressure for 24 h) and obtained in a powder form. All the fractions were dissolved in the cell culture medium (DMEM) and filtered through 0.22 *μ*m filter before being subjected to cell culture treatments. Water insoluble fractions were initially mixed with DMSO before dissolving in DMEM with a final concentration of 0.1% DMSO during cell treatment.

### 2.3. Cell Lines and Culture Conditions

Cancer cell lines MCF-7, HepG2, HCT-15, Hep-2, and A549 and normal cell line MRC-5 were obtained from NCCS, Pune, India. The cells were maintained in DMEM supplemented with 10% FBS at 37°C in CO_2_ incubator in an atmosphere of humidified 5% CO_2_ and 95% air. The cells were maintained by routine subculturing in tissue culture flasks. The culture medium was changed routinely and the cells were split when they reached confluence. The passage number for MCF-7, HepG2, HCT-15, Hep-2, and A549 was P34, P48, P15, P66, and P42, respectively, while performing the cytotoxicity tests.

### 2.4. MTT Assay

This test is based on MTT (3-(4,5-dimethylthiazol-2-yl)-2,5-diphenyl tetrazolium bromide), which is reduced to a purple-blue insoluble formazan precipitate by the living cells. Experiments were performed in six replicates in 96-well flat-bottomed culture plates (Corning). MTT was dissolved in phosphate buffered saline (PBS) at 5 mg/mL. After 24 h of incubation of cancer cells with different concentrations of various fractions of* S. grandiflora*, 20 *μ*L of MTT solution was added and the plate was incubated at 37°C for 4 h. After incubation, 200 *μ*L of dimethyl sulfoxide (DMSO) was added to each well to dissolve the formazan and transferred to fresh microplate. The amount of colored formazan metabolite formed was determined by its absorbance at 570 nm in a VersaMax ELISA Microplate Reader (Molecular Devices Inc., Sunnyvale, CA, USA).

### 2.5. DAPI Staining

Cell nuclear morphology was evaluated by fluorescence microscopy following DAPI staining. A549 cells were treated with the methanolic fraction of* S. grandiflora* for 24 h. The cells were washed with PBS (pH 7.4), fixed with ice cold 70% ethanol and resuspended in DAPI, and incubated for 15 min at 37°C wrapped in aluminium foil. The cells were then washed with PBS and examined under Nikon Eclipse T*i* fluorescence microscope (Nikon Instruments Inc., NY, USA).

### 2.6. Acridine Orange/Ethidium Bromide Staining

Acridine orange/ethidium bromide (AO/EB) staining was carried out to detect morphological evidence of apoptosis. A549 cells were treated with the methanolic fraction of* S. grandiflora* for 24 h. The cells were washed with PBS (pH 7.4) and 10 *μ*L of acridine orange/ethidium bromide solution (60 *μ*g/mL of acridine orange and 100 *μ*g/mL of ethidium bromide in PBS) and made up to 100 *μ*L using PBS and incubated for 5 min. The cells were then washed with PBS and examined under Nikon Eclipse T*i* fluorescence microscope (Nikon Instruments Inc., NY, USA).

### 2.7. Rhodamine 123 Staining

Rhodamine 123 is a fluorescent dye that binds to metabolically active mitochondria. A549 cells were treated with the methanolic fraction of* S. grandiflora* for 24 h. The cells were washed with PBS (pH 7.4) and fixed with ice cold 70% ethanol and incubated with 5 *μ*g/mL rhodamine 123 at 37°C for 30 min. The cells were then washed with PBS and examined under Nikon Eclipse T*i* fluorescence microscope (Nikon Instruments Inc., NY, USA).

### 2.8. DNA Fragmentation Analysis

To confirm the apoptotic mode of cell death, DNA fragmentation assay was performed. A549 cells were treated with the methanolic fraction of* S. grandiflora* for 24 h. After treatment, cells were trypsinized and collected with PBS in 1.5 mL Eppendorf tubes. 100 *μ*L of lysis buffer was added to the pellet and incubated for 30 min on ice. After incubation, centrifugation was carried out at 12,000 g for 30 minutes at 4°C. The supernatant was collected in a fresh tube and mixed with 25 : 24 : 1 mixture of phenol : chloroform : isoamyl alcohol and precipitated with two equivalents of ice cold ethanol and one-tenth equivalent of sodium acetate. This was followed by centrifugation at 12,000 g for 20 minutes. The pellet was resuspended in 30 *μ*L of sterile water-RNase solution (15 *μ*g/mL RNase in sterile water) and 6 *μ*L of 6x loading dye for 30 minutes at 37°C which was electrophoresed and imaged in a Syngene Ingenious gel documentation system (Syngene Bioimaging Pvt. Ltd., Haryana, India).

### 2.9. Western Blot Analysis

Western blot analysis was carried out using cytosolic as well as nuclear fractions [12] of human lung cancer cells A549 treated with selected concentrations of the methanolic fraction of* S. grandiflora*. Protein concentration was determined using Bradford reagent and lysates were resolved on 15% sodium dodecyl sulphate- (SDS-) polyacrylamide gels. The proteins were then electrotransferred onto nitrocellulose membrane (Sigma, St. Louis, MO, USA). After blocking with 5% nonfat milk in Tris-buffered saline (TBS, 0.1 M, pH 7.4), blots were subjected to various primary antibody incubations with mouse monoclonal cyclin D1 antibody, rabbit polyclonal caspase 3 antibody (Santa Cruz Biotechnology, Santa Cruz, CA, USA), and rabbit monoclonal NF*κ*B p65 antibody (Sigma, St. Louis, MO, USA) at 4°C overnight. Protein abundance of *β*-actin and lamin B served as a control for protein loading for cytosolic and nuclear fractions, respectively. Membranes were incubated with secondary antibody, HRP-conjugated goat anti-mouse IgG and HRP-conjugated goat anti-rabbit IgG (Santa Cruz Biotechnology, Santa Cruz, CA, USA), diluted at an appropriate dilution in 1% BSA, for 1 h at room temperature. After each step, blots were washed thrice with Tris-buffer saline-Tween 20 (TBST). Protein bands were detected by enhanced chemiluminescence method (ECL, Bio-Rad, Hercules, CA, USA). The spectral density of the bands was analyzed by Bandscan 5.0 image analyze system. The protein expression pattern was obtained by normalizing the density to that of *β*-actin and lamin B for cytosolic and nuclear fractions, respectively.

### 2.10. Dichlorofluorescein Diacetate (DCF-DA) Staining

ROS levels can be determined by DCF-DA stain. A549 cells were treated with the methanolic fraction of* S. grandiflora* for 24 h. The cells were washed with PBS (pH 7.4) and stained with 10 *μ*M of DCF-DA for 30 min at 37°C wrapped in aluminium foil. The cells were then washed with PBS and examined under Nikon Eclipse T*i* fluorescence microscope (Nikon Instruments Inc., NY, USA).

### 2.11. Analysis Using Spectrofluorometer for Quantification of ROS Generation

A549 cells were treated with the methanolic fraction of* S. grandiflora* for 24 h. Following the treatment, the cells were trypsinized and collected in Eppendorf tubes. 25 *μ*M of DCF-DA was added and incubated for 40 min at 37°C wrapped in aluminium foil. The fluorescence intensity was recorded by using Fluorolog-FL3-11 spectrofluorometer (HORIBA Jobin Yvon, NJ, USA) with excitation and emission wavelengths of 485 nm and 529 nm, respectively, and slit widths set to 5.0.

### 2.12. RNA Isolation and Reverse Transcriptase-PCR

Total RNA isolation was carried out with A549 cells treated with selected concentrations of the methanolic fraction of* S. grandiflora *using TRI reagent (Sigma, St. Louis, MO, USA). The primer sequences used for cyclin D1 and beta actin were previously reported for A549 cells [13] and are as follows:

cyclin D1:

 forward 5′-CCGTCCATGCGGAAGATC-3′;
 reverse 5′-CCTGTCCTACTACCGCCTCA-3′;

*β*-actin:

 forward 5′-AAATCGTGCGTGACATTAA-3′;
 reverse 5′-CTCGTCATACTCCTGCTTG-3′.

### 2.13. Statistical Analysis

All the data were analyzed using the SPSS 7.5-Windows Students version software (SPSS Inc., Chicago, IL, USA). For all the measurements, one-way ANOVA followed by Tukey's test was used to assess the statistical significance between groups. *P* ≤ 0.05 was considered to be statistically significant.

## 3. Results

### 3.1. Effects of* S. Grandiflora* Methanolic Extract on Cell Proliferation

**Figure 1:**
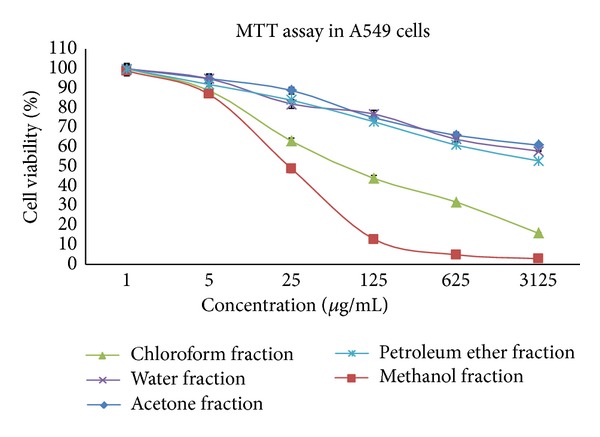
Dose response curve for the effect of the various fractions of* S. grandiflora* leaves in A549 cells for 24 h. Values are mean ± S.D from six independent experiments.

As shown in Table 1, the IC_50_ of* S. grandiflora *methanolic fraction was found to be 23.6 *μ*g/mL in A549 cells which is highly significant compared to the IC_50_ values in other cancer cell lines which include 41.8 *μ*g/mL in HCT-15, 62.7 *μ*g/mL in HepG2, 94.3 *μ*g/mL in MCF-7, and 106.6 *μ*g/mL in Hep-2. The methanolic fraction showed promising anticancer activity with significantly lesser cytotoxicity in normal cells MRC-5 (IC_50_ 104.4 *μ*g/mL) compared to that of the cancer cells. Similar result was observed when the cancer cells were treated with the chloroform fraction but high toxicity was observed even in the normal cells which made it highly unsuitable for our study. Cytoxicity was comparatively much lesser/no cytotoxicity was observed when treated with other solvent fractions such as petroleum ether, acetone, and water. Dose response curve was provided for the effect of various fractions on A549 cells (Figure 1). The regression equation for the effect of methanolic fraction on A549 cells is *y* = − 13.53*ln*(*x*) + 92.768, where *x* is concentration in *μ*g/mL and *y* is the percentage of cell viability. Further analyses were focused on A549 cells with or without the methanolic fraction of* S. grandiflora*.

### 3.2. Effects of* S. Grandiflora* Methanolic Extract on Cellular Morphological Changes of A549 Cells

**Figure 2:**
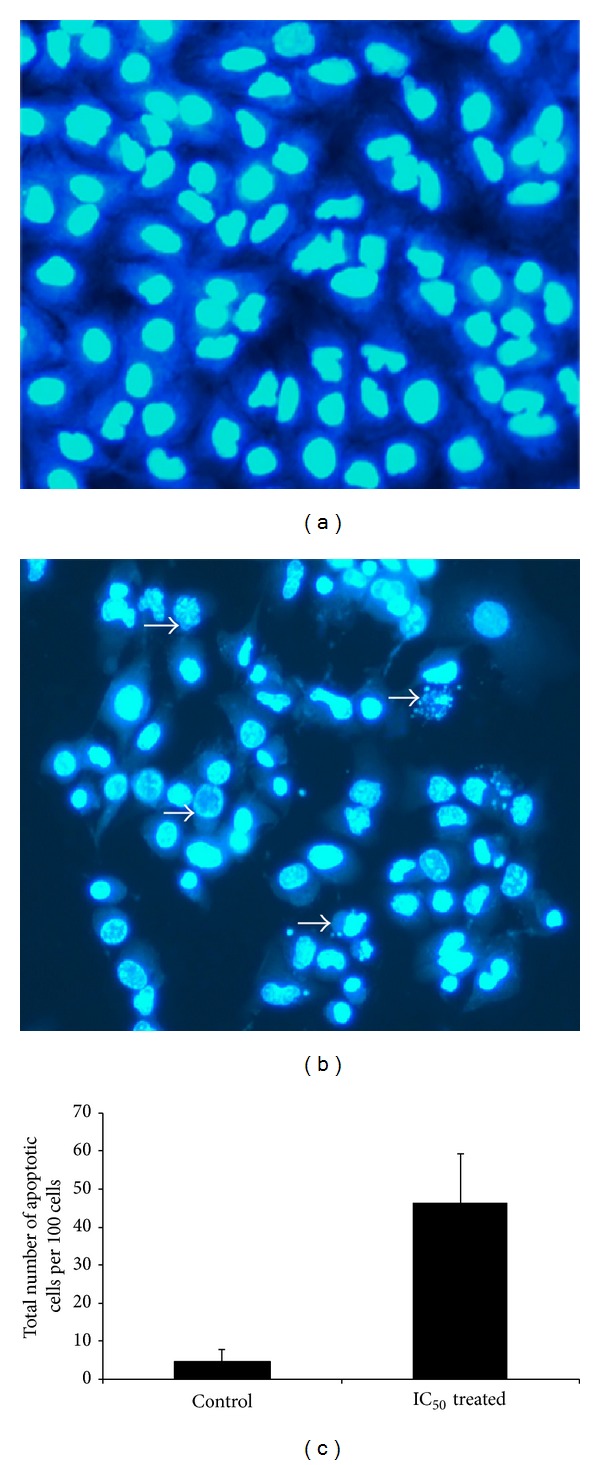
Nuclear staining using DAPI of human lung cancer cells A549 in the presence or absence of the methanolic fraction of* S. grandiflora* leaves. (a) Untreated A549 cells. (b) A549 cells treated with IC_50_ dose of the methanolic fraction for 24 h. Arrows indicate cell shrinkage, nuclear fragmentation, and margination of the nucleus, all associated with the apoptotic mode of cell death. (c) Quantitative results for the number of apoptotic cells per 100 cells in total. Magnification 200x.

**Figure 3:**
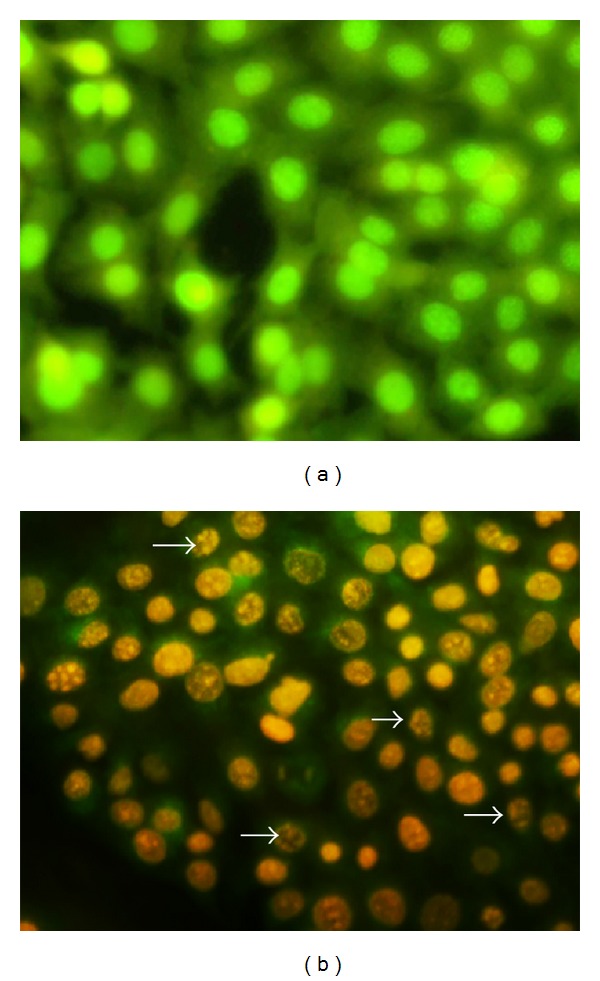
Cellular staining using acridine orange/ethidium bromide of human lung cancer cells A549 in the presence or absence of the methanolic fraction of* S. grandiflora* leaves. (a) Untreated A549 cells. (b) A549 cells treated with IC_50_ dose of the methanolic fraction for 24 h. Arrows indicate cell shrinkage, nuclear fragmentation, and margination of the nucleus, all associated with the apoptotic mode of cell death. Magnification 200x.

We studied the mode of death of A549 cells upon treatment with* S. grandiflora* methanolic fraction. Apoptosis can be differentiated from necrosis by their characteristic nuclear changes. DAPI is a nuclear stain which is observed as blue fluorescence when excited under fluorescence microscope. In our present study, DAPI staining revealed the changes associated with apoptosis in A549 cells treated with the methanolic fraction of* S. grandiflora *(Figures 2(a) and 2(b)). The morphological changes associated with apoptosis such as chromatin condensation, nuclear fragmentation, and margination of nucleus (marked by arrows in Figure 2(b)) are evident in A549 cells upon treatment. Similarly acridine orange/ethidium bromide staining was performed to evaluate the cellular morphological changes in A549 cells treated with the methanolic fraction (Figures 3(a) and 3(b)). Treatment with the methanolic fraction revealed changes associated with apoptosis as indicated by arrows (Figure 3(b)).

### 3.3. Effects of* S. Grandiflora* Methanolic Extract on the Mitochondrial Membrane Potential (ΔΨm) of A549 Cells

**Figure 4:**
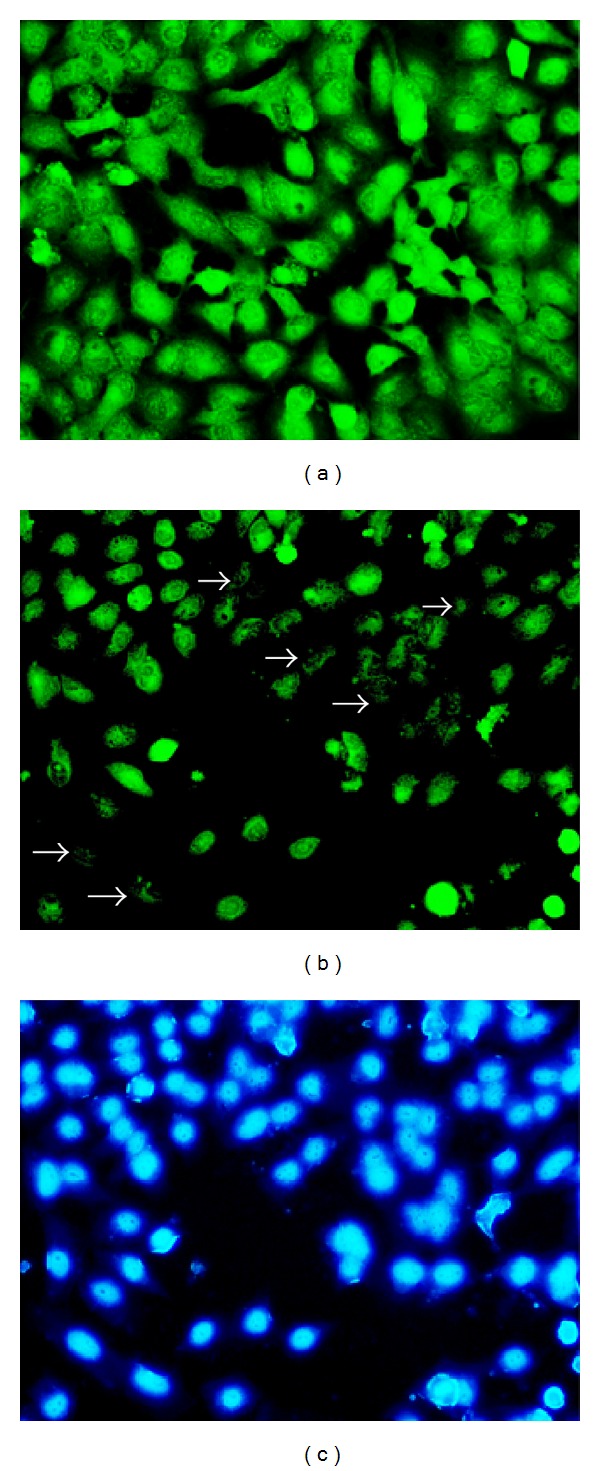
Mitochondrial staining using rhodamine 123 of human lung cancer cells A549 in the presence or absence of the methanolic fraction of* S. grandiflora* leaves. (a) Untreated A549 cells. (b) A549 cells treated with IC_50_ dose of the methanolic fraction for 24 h showing decreased membrane potential as indicated by the arrows. (c) DAPI costaining of A549 cells treated with IC_50_ dose of the methanolic fraction. Magnification 200x.

Mitochondrial membrane potential can be evaluated by staining with rhodamine 123. Green fluorescence is observed in cells with high membrane potential. We found that, upon treatment with the methanolic fraction, the mitochondrial membrane potential was decreased in A549 cells as evidenced by the decrease in the fluorescence compared to the untreated cells (Figures 4(a) and 4(b)). DAPI was used as a counterstain to locate the cells with decreased membrane potential (Figure 4(c)).

### 3.4. Apoptosis Confirmation by DNA Fragmentation

**Figure 5:**
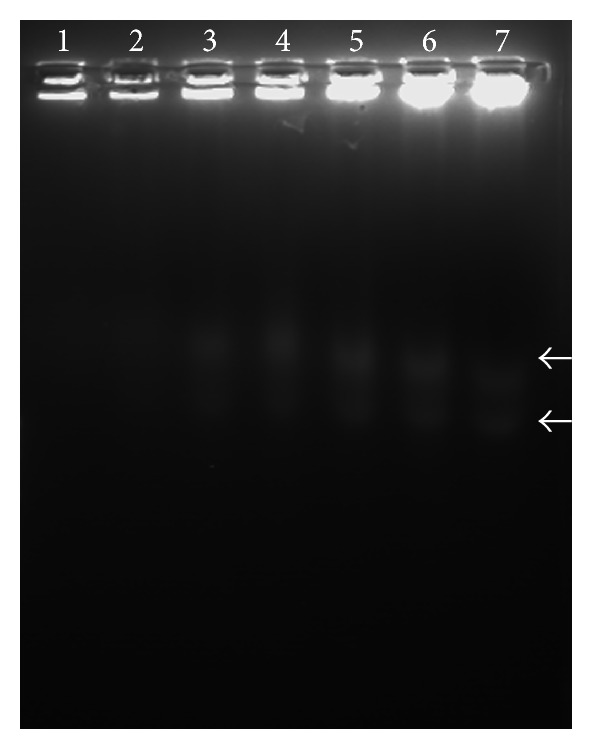
DNA fragmentation analysis for human lung cancer cells A549 in the presence or absence of the methanolic fraction of* S. grandiflora* leaves. Lane 1 is untreated A549 cells. Lane 2 is 5 µg/mL treated. Lane 3 is 10 µg/mL treated. Lane 4 is 15 µg/mL treated. Lane 5 is 20 µg/mL treated. Lane 6 is 25 µg/mL treated. Lane 7 is 30 µg/mL treated. All the treatment is for 24 h.

To gain further insights into the mode of cell death caused by* S. grandiflora *methanolic fraction, we determined its effect on the DNA fragmentation, a widely used technique for the detection of apoptosis. The treatment resulted in a dose-dependent increase in the DNA fragmentation levels in A549 cells (Figure 5). DNA fragmentation was clearly visible in the groups with the dose-dependent treatment of the methanolic fractions (Lanes 2 to 7) compared to the control group (Lane 1).

### 3.5. Protein Expression of Caspase 3, Cyclin D1, and NF*κ*B p65

**Figure 6:**
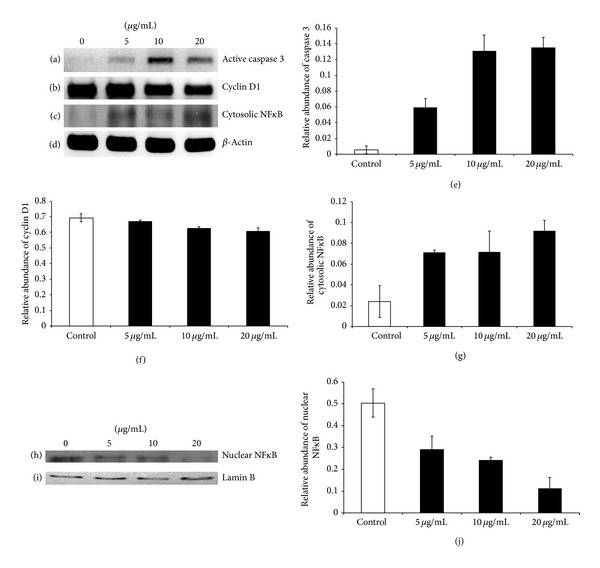
Western blotting analysis for the protein expression of caspase 3 and cyclin D1 from human lung cancer cells A549 in the presence or absence of the methanolic fraction of* S. grandiflora *leaves. (a) Protein abundance of active caspase 3. (b) Protein abundance of cyclin D1. (c) Protein abundance of cytosolic NF*κ*B. (d) Loading control *β*-actin. (e) Densitometry for the protein abundance of active caspase 3. (f) Densitometry for the protein abundance of cyclin D1. (g) Densitometry for the protein abundance of cytosolic NF*κ*B. (h) Protein abundance of nuclear NF*κ*B. (i) Loading control lamin B. (j) Densitometry for the protein abundance of nuclear NF*κ*B. Values are mean ± S.D from three independent experiments. ANOVA followed by Tukey's test was used to assess the statistical significance between groups. *P ≤ 0.05, significance relative to control.

To confirm the results of* S. grandiflora* induced apoptosis in A549, we further investigated whether increased caspase 3 activity was observed through western blotting. As predicted, a dose-dependent increase in the caspase 3 expression was observed (Figure 6(a)). Densitometry of caspase 3 expression showed that there was a significant increase in the levels of caspase 3 starting at 1/4  IC_50_ dose treatment of methanolic fraction (Figure 6(e)). Since cell cycle plays a major role in many instances of cancer, we planned to evaluate the expression levels of cyclin D1 which helps in the cell cycle G1/S transition. We observed that there was a dose-dependent decrease in the expression levels of cyclin D1 when A549 cells were treated with* S. grandiflora* methanolic fraction (Figure 6(b)). Densitometry of cyclin D1 expression showed that there was a significant decrease in the levels of cyclin D1 (Figure 6(f)). We also found that the cytosolic abundance of NF*κ*B increased with the treatment of the methanolic fraction (Figures 6(c) and 6(g)). Nuclear abundance of NF*κ*B decreased with the treatment of the methanolic fraction (Figures 6(h) and 6(j)). The protein expression levels of *β*-actin were used as the loading control for cytosolic fraction (Figure 6(d)) and lamin B was used as the loading control for the nuclear fraction (Figure 6(i)).

### 3.6. Evaluation of Intracellular Reactive Oxygen Species (ROS)

**Figure 7:**
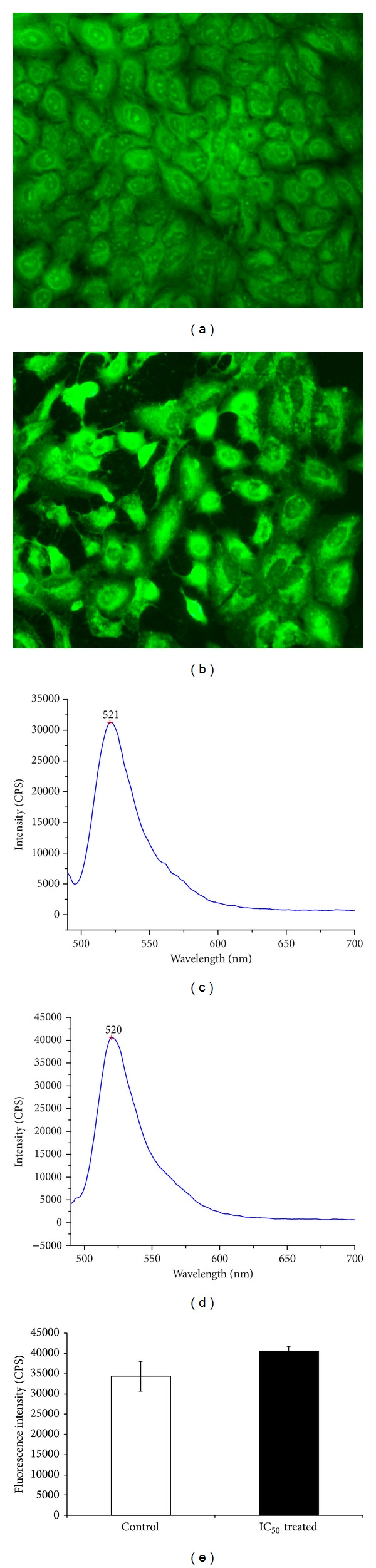
DCFDA staining for ROS generation of human lung cancer cells A549 in the presence or absence of the methanolic fraction of* S. grandiflora* leaves. (a) Untreated A549 cells. (b) A549 cells treated with IC_50_ dose of the methanolic fraction for 24 h. (c) Spectrofluorometer measurement for the levels of ROS intermediates in untreated A549 cells. (d) Spectrofluorometer measurement for the levels of ROS intermediates in treated A549 cells. (e) Densitometry graph comparing the levels of ROS intermediates in untreated and treated A549 cells. Values are mean ± S.D from three independent experiments. ANOVA followed by Tukey's test was used to assess the statistical significance between groups. *P ≤ 0.05, significance relative to control.

ROS generation was usually associated with cell apoptosis. To compare the level of ROS generation in A549 cells with or without the methanolic fraction of* S. grandiflora*, we used an oxidation-sensitive fluorescent dye, 2′, 7′-dichlorofluorescein diacetate. Fluorescent microscopy images revealed the increased generation of ROS in the treatment group (Figure 7(b)) compared to that of control (Figure 7(a)). A significant increase in the levels of dichlorofluorescein fluorescence was detected in the treated cells compared to control as evidenced by spectrofluorometer readings (Figures 7(c), 7(d), and 7(e)).

### 3.7. Gene Expression of Cyclin D1

**Figure 8:**
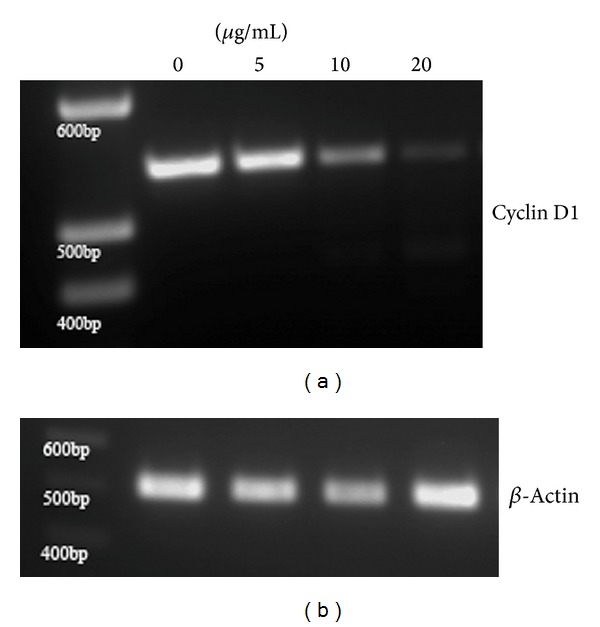
Reverse transcriptase PCR for the gene expression of cyclin D1 from human lung cancer cells A549 in the presence or absence of the methanolic fraction of* S. grandiflora *leaves. (a) Gene expression of cyclin D1. (b) Loading control *β*-actin.

In order to evaluate whether the decrease in cyclin D1 expression occurred at the translational level or transcriptional level, reverse transcriptase-PCR was performed. The gene expression of cyclin D1 decreased with the treatment of the methanolic fraction (Figure 8(a)) indicating that the cyclin D1 was decreased at the transcriptional level. The gene expression of beta actin served as the loading control (Figure 8(b)).

## 4. Discussion

The use of naturally occurring plant-based products has shown promising results in the treatment of cancer. One renowned example is the use of taxol, a plant-based bioactive compound used in cancer chemotherapy [14]. Due to the complexity of cancer, novel bioactive compounds with multitargeting efficacy are the need of the hour. We tried to evaluate the anticancer potential of a traditional plant, used as a medicine in India and most parts of Southeast Asia, called* Sesbania grandiflora*. Our present findings demonstrate that the methanolic fraction of* S. grandiflora *has potent anticancer activity against human lung cancer cells and comparable activity in few other cancer cell models while significant cytotoxicity was not observed in the normal cells. One possible reason could be the plant's modulatory effect on apoptosis, which is usually blocked in cancer cells, thereby promoting cell death faster compared to that of normal cells.

Evaluation of apoptosis is crucial to differentiate it from necrosis. Nuclear fragmentation is one of the characteristic features of apoptotic mode of cell death. We used DAPI, a fluorescent DNA-binding agent, and acridine orange/ethidium bromide staining to observe cell death and cellular morphological changes involved in apoptosis [15]. The treatment group showed fragmented apoptotic bodies, shrunken and marginated nuclei in contrast to the normal and large nucleus in the untreated cells, proving the apoptotic potential of the extract. Decrease in mitochondrial membrane potential usually indicates apoptosis and helps to distinguish the mode of cell death from necrosis [16]. Our findings confirmed the decrease in the mitochondrial membrane potential as evidenced from the decreased rhodamine 123 fluorescence intensity when A549 cells were treated with the IC_50_ dose of* S. grandiflora*.

DNA laddering is one of the hallmark indications that helps in the differentiation of apoptosis from necrosis [17, 18]. In the treatment group of our present study, the presence of the laddering pattern of DNA fragments and the absence of the necrotic streak further confirm apoptosis. DNA laddering occurs through caspase-activated DNases (CAD) which is activated by caspases. In order to confirm the involvement of caspases in our study, we evaluated the protein expression levels of caspase 3, the common caspase for most of the death signals. As predicted, there was a dose-dependent increase in the levels of active caspase 3.

Arrest of cell cycle can also trigger apoptosis. Cyclins are the proteins which regulate the cyclin-dependent kinases, whose activity in turn regulates the cell cycle checkpoint transitions. One such cyclin, cyclin D1, is involved in the transition of cells from G1 to S phase of the cell cycle. Overexpression of cyclin D1 has been reported in many cancers [19–21] and it was shown that the inhibition of cyclin D1 expression could help in the cancer treatments [22, 23]. In several studies, it was observed that the decrease in cyclin D1 levels is associated with apoptosis [24, 25]. In our present study, we observed a decrease in the expression levels of cyclin D1 which could have probably mediated the antiproliferative and apoptotic effects of the plant.

In order to determine the upstream signal transduction effectors for the possible mechanism of action for the plant's modulatory effect on cell cycle and apoptosis, we focused on the transcription factors that might regulate them. One of the important transcription factors is nuclear factor kappa B (NF*κ*B) which has been implicated in cell proliferation as well as tumor development. NF*κ*B is present in the cytosol in an inactive form tightly bound with cytoplasmic I*κ*B proteins [26]. Activated NF*κ*B by the release from I*κ*B proteins translocates into the nucleus to activate the transcription of the target genes. Activated NF*κ*B has been demonstrated to be involved in the prevention of apoptosis [27] as well as in the progression of cell cycle of G1 to S transition through regulation of cyclin D1 [28]. This gives an overall idea regarding the activation of caspase 3 as well as decrease in the protein abundance of cyclin D1. In our present study, the levels of NF*κ*B p65 in the cytosol increased with the concomitant decrease in the nucleus of A549 cells after treatment with the methanolic fraction, thereby preventing the activation of several target genes including cyclin D1. In order to verify the inhibition of cyclin D1 expression at the transcriptional level, RT-PCR was performed which confirmed that the decrease in the cyclin D1 expression occurs at the transcriptional level which correlates with the decreased NF*κ*B p65 activation. Thereby the mechanism of action of the methanolic fraction of* S. grandiflora* could be associated with a pathway which prevented the activation of NF*κ*B.

**Figure 9:**
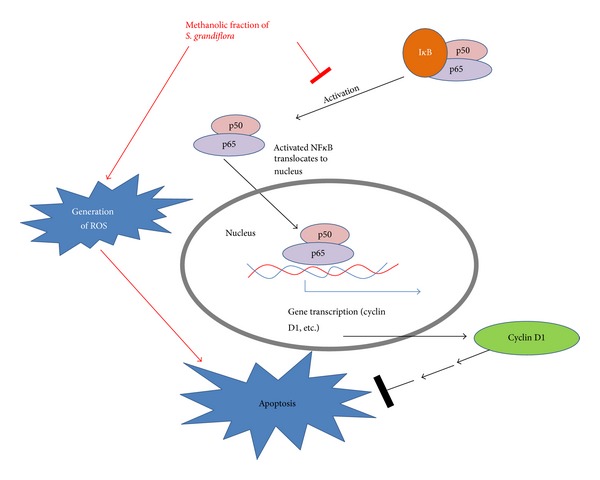
Schematic illustration.

Apoptotic induction can also be mediated through ROS intermediates [29–31]. It was reported that ROS could play a role in downregulating Bcl-2 [32] as well as in triggering the release of cytochrome C from the mitochondria into the cytoplasm along with Fas associated proteins recruitment and finally leading to the activation of caspase 3 and apoptosis [33–35]. In our present study, increased levels of ROS in the treatment group indicated that the apoptotic mode of cell death induced by the methanolic fraction of* S. grandiflora *is through the generation of ROS. Thereby, an outline to the mechanism of action for the anticancer effect of* S. grandiflora* leaves involves the prevention of the transcription of major genes by inhibiting NF*κ*B activity and arresting the cells in G1/S phase as well as the generation of ROS intermediates, all triggering the apoptotic cascade leading to cell death (Figure 9).

These observations clearly suggest that the antiproliferative effect exerted by the leaves of* S. grandiflora* is associated with apoptosis. A novel source for a cytotoxic drug is thus warranted from our current findings. Most secondary metabolites from plants have a complex architecture and are synthesized with the involvement of a wide range of enzymes. This explains in part the multiple target efficacy exerted by plant-based products.* S. grandiflora* indeed could harbor such molecules with multiple target efficacies. Further study on the structural elucidation of the active components responsible for the anticancer activity as well as the detailed molecular mechanism of action to reveal the upstream effectors of apoptosis is in progress.

## 5. Conclusion

From the results, it is clear that the alcoholic extract of* S. grandiflora* exerted antiproliferative effects especially on lung cancer cells. The mode of cell death of cancer cells by* S. grandiflora* predominately followed apoptosis. A possible G1/S arrest as confirmed through decreased cyclin D1 expression might have triggered apoptosis. The mechanism of action of the methanolic extract in lung cancer cells could possibly involve a pathway that prevents NF*κ*B activation. Thus, the results of this study have led to a new source of plant exerting potent antiproliferative and apoptotic effects. Our present study also suggests the possibility of a lifesaving drug combating different types of cancers especially lung cancer.

## Figures and Tables

**Table 1:** IC_50_ values for the cytotoxicity of various solvent fractions from the leaves of *S. grandiflora *determined using MTT assay.

	IC_50_ (µg/mL)					
	MCF-7	HepG2	A549	HCT-15	Hep2	MRC-5
Petroleum ether fraction	>500	>500	>500	>500	>500	>500
Chloroform fraction	116.8	93.2	74.1	52.3	92.6	104.3
Acetone fraction	332.7	473.9	>500	>500	388.1	>500
Methanol fraction	94.3	62.7	23.6	41.8	106.6	104.4
Water fraction	>500	>500	>500	>500	>500	>500
